# Radioactive iodine and female fertility

**DOI:** 10.1038/s41598-022-07592-8

**Published:** 2022-03-08

**Authors:** Pino Navarro, Sandra Rocher, Pau Miró-Martínez, Sandra Oltra-Crespo

**Affiliations:** 1 Department of Endocrinology, Hospital Virgen de los Lirios Alcoy, 03804 Alicante, Spain; 2 Department of Endocrinology, Bernabéu Institute of Reproductive Medicine, 03016 Alicante, Spain; 3 Department of Gynaecology and Obstetrics, Hospital Reina Sofía, 30003 Murcia, Spain; 4 Department of Gynaecology and Obstetrics, Hospital Universitario Virgen de la Arrixaca, 30120 Murcia, Spain; 5 Department of Applied Statistics and Operational Research and Quality, Universitat Politècnica de València, Valencia, Spain; 6 Department of Applied Mathematics, Universitat Politècnica de València, Valencia, Spain

**Keywords:** Endocrine cancer, Thyroid cancer, Cancer therapy, Endocrinology, Endocrine system and metabolic diseases, Endocrine reproductive disorders, Reproductive disorders, Infertility

## Abstract

Radioactive iodine (I^131^) is used after surgery in the treatment of Differentiated Thyroid Carcinoma (DTC). There is no solid evidence about the potential deleterious effect of I^131^ on women fertility. The objective of this study is to assess the impact that I^131^ may have on fertility in women. All women followed by DTC in our department have been analyzed and women younger than 45 years old at the time of diagnosis and initial treatment were included. There were 40 women exposed to I^131^ (study group) and 11 women who were only treated with thyroidectomy (control group). Of the women exposed to I^131^, 40% went through early menopause, while no cases were reported among their controls. Furthermore, 29.2% of women exposed to I^131^ had decreased Antimüllerian Hormone (AMH), compared to the only 11% of unexposed women (not significant). Regarding the fertility impairment "perceived" by patients, in the group of women exposed to iodine, 17.9% described being unable to complete their genesic desire whereas, none was registered in the control group. We conclude that radioactive iodine can affect a woman's fertility and shorten her reproductive life, so this is an aspect that should be taken into consideration.

## Introduction

Thyroid cancer is the most frequent endocrine malignancy. Differentiated tumours derived from the follicular epithelium, papillary (85%) and follicular carcinoma (15%), account for around 90% of thyroid tumours and fortunately, are the ones with the best prognosis. Papillary carcinoma has two age peaks of presentation: between the second and third decade of life, followed by a second later peak. Follicular carcinoma usually appears in older individuals. In this type of cancer, the female/male relation reaches a ratio of approximately 3.5/1^1^. Given its good prognosis, it is expected to have a good quality of life after the treatment and achieve a life expectancy similar to that of the rest of the population; that is why the possible effects derived from the treatment of the disease, including those related to future reproductive health, must be assessed.

Radioiodine treatment in low-risk patients is currently a matter of discussion. In these patients, the prognosis is excellent, mortality is low and the controversy resides in whether iodine really provides benefits in terms of recurrence^2,3^. The treatment with radioactive iodine is not innocuous since the uptake is not only at thyroid level and during its elimination the radiation is discouraged towards other tissues. The most frequent complication is sialoadenitis due to iodine uptake by the salivary glands. Transitory loss of flavours perception, buccal dryness and dental caries, nasolacrimal duct stenosis and even pulmonary fibrosis and radiation neumonitis could also appear. Complications, despite being rare, usually appear in patients with a high treatment load. A statistically significant increase in second malignancies has been reported closely related to the administered dose. The secondary most related neoplasias are leukaemia, salivary gland, breast, digestive tract, and bone cancer. Despite this, the overall risk is low^4^. Another adverse effect is the impairment of fertility^5,6^.

In men, I^131^ therapy is associated with a temporal increase in FSH and Inhibin B decrease, being related serum FSH with the received dose. The alteration of these values shows the damage to the germinal epithelium that results in azoospermia and oligospermia. On the other hand, damage to Leydig cells manifests itself as an elevation of LH levels and testosterone lowering. The effect on the male gonad responds to a dose-dependent effect. While low doses of iodine do not have negative consequences for reproduction, only transient alteration of hormone levels and recovery is the rule, cumulative doses greater than 500–800 mCi raise the risk of permanent increases in FSH, which could lead to infertility^7,8^.

In women, studies show short and long-term consequences for fertility due to the iodine treatment. I^131^ is associated in the short term with menstrual disorders that occur in the first year after iodine administration. Around 20–30% of women go through a period of amenorrhea or metrorrhagia that occurs with at least one menstrual cycle of latency and whose duration would not exceed a year^9,10^. Later studies find similar results and emphasize that the temporary absence of menses would occur more frequently in women with older ages^11,12^. Another fact to support the damage of iodine to female fertility is the finding of the age of menopause advancement in women treated with I^131^ ^13^, which would imply a decrease on their fertile period. In 2015, the study by James Wu et al. uses a retrospective cohort of 18,000 women to demonstrate that patients with differentiated thyroid carcinoma who are treated with I^131^ have a decreased birth rate compared to patients who are not administered this therapy, and that this phenomenon occurs, especially, in women who are treated at later ages^14^. This study leads us to believe that the decrease in births could be due to the harm of I^131^ to the ovarian reserve. To approximate the ovarian reserve, the most accurate and sensitive parameter due to its low inter and intracycle variability is the determination of the Antimüllerian Hormone. AMH is produced in the granulose cells of growing ovarian follicles, especially in preantral and small antral follicles, preventing the development of other smaller follicles. AMH levels show a progressive decrease throughout the reproductive life as the follicular reserve declines, finally becoming undetectable shortly before menopause^15,16^. Despite having established itself as a relevant marker, there is little data in the literature on normal AMH values in healthy population, compared to women who attend fertility clinics. One of them is the article by La Marca et al., which establishes a nomogram of AMH values with a large population of healthy women^17^. F. Acibucu et al. in 2016 analyzed the potential effect of iodine on fertility, using the antimüllerian hormone for the first time as indicator of the ovarian reserve. The study found significantly lower mean AMH values in women who were exposed to iodine^18^. Giusti et al. in 2018 also used the AMH to assess the effect of iodine in a population of 34 women treated with I^131^ versus 23 women without exposure with an average follow-up period of 7 years. Although lower AMH values were found in exposed women, the results do not obtain statistical significance, probably due to the sample size^19^. In 2018 I Yaish et al. demonstrated how a single dose of radioactive iodine has an effect, on ovarian reserve, estimated through AMH before and after exposure over a year. The results describe in patients treated with higher doses of I^131^ a significant decrease in the concentration of AMH, with the nadir occurring at 3 months, when the values are almost 50% lower than the prior levels to treatment. Subsequently, an incomplete recovery is seen towards the end of the first year that stagnates at around 9 months, at which time the AMH values are 32% lower than the initial ones^20^. This finding is confirmed in other studies such as the one published by B Evranos in the same year^21^, or the most recent of EFS van Velsen published in 2020^22^. However, all these recent works are limited to the study of ovarian reserve after receiving radioiodine treatment, but do not evaluate the long-term consequences on the impact on the duration of the reproductive life years and on the achievement of the objectives of desire for offspring.

The aim of this study is to assess the impact that I^131^ can have on fertility in women in our patients’ population with differentiated thyroid carcinoma. For this purpose, we study the age of menopause if it has already taken place at the time of the study or the Antimüllerian hormone if women still have their periods and assess the achievement of their reproductive desire.

## Methods

### Study population

From the entire population of women monitored for Differentiated Thyroid Carcinoma by the Health Department of Alcoy, just women with ages less or equal to 45 years at the time of initial diagnosis and treatment are included in the study. Patients are classified based on the administration of radioactive iodine as part of treatment in addition to surgery. Women treated with surgery and I^131^ are the cases and women treated only with surgery without exposure to iodine are their controls.

Currently 87 women are being monitored by the Endocrinology Department for having suffered from DTC. Thirty-four women were excluded from the study because they were diagnosed being older than 45 years of age and 2 more women because they were already menopausal at the time of diagnosis.

Fifty-one women with history of DTC aged less or equal to 45 years at the time of initial diagnosis and treatment were included in the study. None of the 51 women included had a history of previous pelvic radiotherapy or surgery.

### Study protocol

Fertility is studied by assessing the age of menopause onset if it has already occurred, the Antimüllerian Hormone (AMH) in menstruating women at the time of the study, and analysing the obstetric history and the reproductive desire of patients.

Through the medical history and the follow-up, data such as characteristics of the menstrual cycles, age of menopause, pregnancies, miscarriages, use of assisted reproductive techniques, and the appearance of second malignancies are collected.

The usual analysis of the patients included the determination of AMH if they still had menses and FSH and estrogens in women without menstrual cycles.

A review of available reports about pathological anatomy of surgical pieces allowed us to describe the histological variant and its characteristics. Likewise, a genetic study of BRAF mutations was performed in papillary carcinomas and KRAS in follicular carcinomas for which we had a sample of sufficient size.

### Valuation criteria

#### Spontaneous menopause

Women who have had a hysterectomy during follow-up have been excluded from this evaluation. It is considered early menopause when it appears at an age below 45 years old and it has a compatible hormonal analysis.

#### Decreased ovarian reserve

Determination of AMH in women who have not yet undergone spontaneous menopause, although they have undergone a hysterectomy without associated oophorectomy and who do not have a history of polycystic ovary syndrome. As AMH prior to radioiodine treatment was not available, the post-iodine AMH value is compared to the AMH nomogram published by La Marca et al^17^. Patients have been considered to have a compromised ovarian reserve when their AMH value is below the 5th percentile of the AMH nomogram.

#### Reproductive desire fulfilment

It is evaluated from data of the obstetric history and the own evaluation of each woman about the fulfilment of her reproductive desire. The need for the use of Assisted Reproduction Techniques (ART) has also been assessed.

### Statistical analysis

For continuous variables, mean, standard deviation, median and distribution by quartiles are used. Normality tests are performed using Kolmogorov–Smirnov test on samples with more than 50 records and Shapiro–Wilk test on smaller samples. For means comparison on continuous variables, Student's T test was used in those samples with normal distribution and Mann–Whitney’s U test was used in variables that did not meet normality criteria. For categorical variables, percentages are used. In the frequency comparison, the exact Fisher statistic is used since it is not possible with all parameters under study to collect a minimum of 5 expected frequencies in all cells. Odds Ratio (OR) calculation is performed to determine the risk supposed by exposure of radioactive iodine to produce a decreased ovarian reserve, early menopausal age, and impaired fertility. To assess the relationship between variables, Spearman correlation coefficient is used, since the studied variables do not follow a normal distribution. Since menopause is a unique and irreversible event in a woman's life, the age of menopause is also studied using a Kaplan–Meier survival analysis. To perform all the statistical analysis SPSS platform was used.

### Ethical considerations and Informed consent

All procedures performed were in accordance with the ethical standards of the institutional and/or national research committee and with the 1964 Helsinki declaration and its later amendments or comparable ethical standards. All subjects gave written consent to participate. All procedures were evaluated and approved by the General University Hospital of Elche Committee on human experimentation, in accordance with the 1975 Helsinki Declaration. Informed consent to inclusion in the study was obtained from all patients.

## Results

### General characteristics of the study population

The study population is made up of 51 patients with a mean follow-up time of 12.6 years. Characteristics of study participants are shown in Table 1.

**Table 1:** Characteristics of study participants: age at diagnosis and age at the study, follow-up time and tumour characteristics.

	Total	I^131^ treatment	Controls	*p*
No. patients	51	40	11	
Initial age^a^				
Years (mean ± SD)	30.8 ± 8.3	29.6 ± 8.2	35.2 ± 7.9	0.048
Range	15.4–44.9	15.4–44.9	22.1–44.4	
Final age^b^				
Years (mean ± SD)	43.5 ± 9.2	39.7 ± 6.8	42.7 ± 5.5	0.856
Range	24.3–65.3	24.3–65.3	29.7–55.6	
Follow up period				
Years (mean ± SD)	12.6 ± 9.8	13.7 ± 9.7	8.7 ± 9.7	0.135
Median	12.5	14.3	4.3	
Range	0.5–36.8	0.6–36.8	0.5–25.6	
Microcarcinoma (< 1 cm)^c^	12/47 (25.5%)	4/36 (11.1%)	8/11 (72.7%)	0
Low Risk (ATA)^d^	34/51 (66.7%)	23/40 (57.5%)	11/11 (100.0%)	0.009
BRAF+	11/20 (55.0%)	10/18 (55.6%)	1/2 (50.0%)	–
KRAS+	1/3 (33.3%)	1/3 (33.3%)	0/0 (0.0%)	–

^a^Age at diagnosis and start treatment.

^b^Age at the time of present study.

^c^In 4 cases we do not know the tumour size.

^d^ATA (American Thyroid Association) risk classification.

As we see, women who have received iodine treatment are significantly younger at the time of initial diagnosis and treatment. As expected, they have larger tumours and are at higher risk according to the ATA (American Thyroid Association) classification.

Inside the group of women who were only treated with surgery, all had histological diagnosis of papillary thyroid carcinoma and all of them were classified as low-risk patients. There were microcarcinomas in 72.7% of the cases. In the group of I^131^ exposed patients, 57.5% are considered of low risk, 37.5% intermediate risk and 5% high risk, one of the cases due to the presence of lung metastases and the other due to extensive local extension. There are 37 papillary carcinomas (92.5%) and 3 follicular carcinomas (7.5%). Regarding the size of the tumors, only 4 (11.1%) are microcarcinomas.

The study of genetic mutations reveals a positivity of 50–55% for the BRAF mutation in papillary carcinomas and 33.3% in follicular carcinomas.

When we study the dose used to treat DTC, almost 70% of women receive high doses of iodine (> 100 mCi). Only 15% of patients received less than 50 mCi. Radioiodine dosage is described in Supplementary material Table 1.

It has also been reported some menstrual alterations after radioiodine treatment among our patients. The frequency of these irregularities distributed by dosage is shown in Supplementary material Table 2.

**Table 2:** Cases of women with menopause classified by age of onset before or after 45 years.

	I^131^ treatment	Controls	*p*
Spontaneous Menopause N (%)	10/36 (27.8%)	1/10 (10%)	0.400
Menopause mean age	44.6 ± 1.857	49	–
Early menopause (< 45 years old) N (%)	4/10 (40%)	0/1	1.000

### Menopause age

Women undergoing hysterectomy are excluded from the analysis (four in the group exposed to radioactive iodine and one in the control group).

Eleven women (23.9%) showed menopause. The frequency of menopausal cases distributed according to age is shown in Table 2.

**Figure 1:**
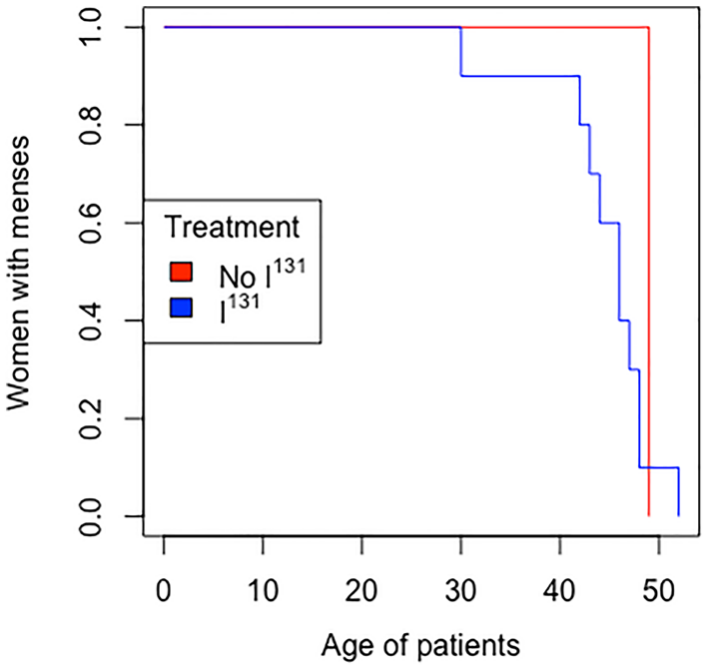
Kaplan–Meier graphic for menopausal women in I^131^ exposed group and their control.

When comparing the frequency of spontaneous menopause between both groups, there are no significant differences between the women exposed to iodine and their controls. Similarly, given the scarcity of cases, we cannot speak of statistically significant differences in the appearance of early menopause in our series of patients. However, there seems to be a trend towards early menopause in women exposed to I^131^ (Fig. 1).

### AMH alteration

When we compare mean AMH level in the radioiodine group and the control group, regardless of the age, there are no significant differences (Table 3).

**Table 3:** Summary of results of the AMH levels study.

	I^131^ treatment	Controls	*p*
AMH (ng/ml)	1.18 ± 1.2	1.24 ± 1.0	0.692
AMH < P5	11/30 (36.7%)	2/10 (20%)	0.451

**Figure 2:**
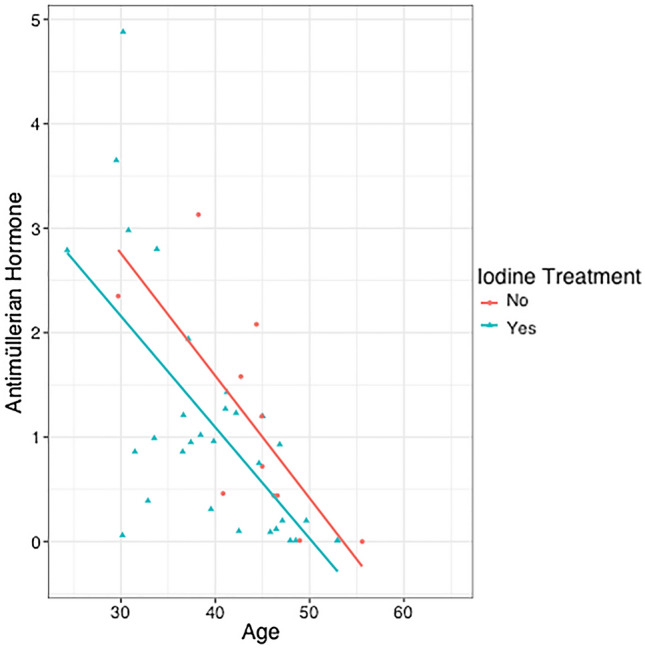
Correlation of AMH values and age of women exposed to iodine and controls.

Once deeply studied the AMH level behaviour, we see there is always a negative correlation between age and AMH level (Fig. 2).

The relationship is statistically significant for both groups, exposed women (*p* = 0.001) and the control group (*p*-value = 0.002). What varies is the strength of the association, in the control group, Spearman’s Rho statistic is -0.851 (strong association) and in the group of exposed women, Spearman's Rho is -0.582 (moderate association).

When comparing the individual AMH values with the proposed nomogram of La Marca et al.^17^ it is obtained that there are 13 patients whose value is below the 5th percentile. Eleven of those women with low ovarian reserve belong to the iodine exposure group. Only 2 patients with decreased AMH values have not been treated with radioactive iodine (Table 3).

The measure of the association between decreased AMH and I^131^ exposure gives an Odds Ratio of 2.3158. However, it does not turn out to be statistically significant (CI: 0.4514–12.9097).

### Impairment of fertility

Among women in our study, 8 expressed to have failed to fulfil their reproductive desire. This represents 16% of the sample. One of the women who considered their fertility to be affected belongs to the control group, while the remaining 7 women were exposed to radioactive iodine.

The woman who belongs to the control group, prior to the diagnosis of thyroid carcinoma, had already consulted for sterility, therefore we cannot implicate the carcinoma in the impairment of fertility in this case. This means that there are 7/40 women whose fertility was affected after the diagnosis, and all of them were exposed to I^131^, or what is the same, 17.5% of the women who were treated with I^131^ consider to have not achieved their reproductive desire.

The difference in frequency of fertility impairment according to I^131^ exposure was not statistically significant. The same as the association measure (OR) calculation whose confidence interval is 0.239–19.987.

## Discussion

The data presented, although not conclusive, should raise alarms among specialists who face differentiated thyroid carcinoma. This study includes a small patient sample, similar to the studies found in recent literature^9–14,18–21^.

Regarding the advancement of menopausal age, our results are relevant. Firstly, it is observed that women who belong to the group of patients exposed to radioactive iodine are significantly younger at the time of initial diagnosis and treatment, on average 29.6 years old, while their controls are on average 35.2 years old. On the other hand, all registered cases of early menopause are in women who have received radioactive iodine treatment and, while in the general population the prevalence of early menopause reaches an approximate percentage of 10%^23^, among women who have been treated with I^131^ in the study, this percentage rises to 40%. In the population of Alcoy, the age for searching the first child in 2016 is estimated at 30 years^24^. The advancement of the age of menopause reduces even further the reproductive period of women who today plan to search for pregnancy at increasingly later ages. The study by Ceccarelli et al.^13^ in 2001 shows data and graphs similar those of this work. When comparing the menopausal age in women with DTC treated with I^131^ and patients with goiter, we find a significantly earlier age of menopause onset in women with cancer. We agree that this phenomenon could be due to iodine-induced ovarian damage. This damage seems to contribute to the decline in ovarian function and hasten the process of follicular atresia in premenopausal women, especially those with a reduced pool of viable follicles.

Antimüllerian hormone is recognized as the most robust ovarian reserve marker available^15^. AC de Kat et al.^25^ clarifies that the AMH estimates the reproductive period of women regardless of chronological age. In other words, that a woman with a low AMH level will have a lower ovarian reserve and therefore a shorter period of time until menopause than a woman of the same chronological age with higher AMH values. However, in all women, over the years, the reserve decreases and with it, the antimüllerian hormone also diminishes. The speed of descent is not the same in all women, as it is also affected by genetic dotation and damage accumulation, but we can establish a correlation between age and AMH value with a powerful association. In our work, we corroborate this fact by correlating the age and AMH values of our patients. The result in the case of women from control group is a negative and significant correlation that shows a strong association between the variables. However, this correlation, in the case of women exposed to I^131^, continues to be significant but it is distorted and the association strength goes from being strong to moderate. This tells us that despite not finding differences in the mean AMH value between the exposed group and the control group, I^131^ effectively leaves an indelible mark on the ovarian reserve.

The final consequence of the menopause age advancement and the decline of the ovarian reserve is the difficulty in obtaining spontaneous pregnancies and the non-fulfilment of the reproductive desire. In this work, there are 17.5% women exposed to I^131^ who report not having achieved their genesic desire. Among these women, there are 3 affected by early menopause, one of them started with the typical signs and symptoms of menopause immediately after treatment at the age of 28; Three women with a highly compromised ovarian reserve, who had repeated abortions before pregnancy and one of them was forced to resort to assisted reproduction techniques to achieve pregnancy; and a last woman who had a very intense metrorrhage after treatment with I^131^ and underwent a hysterectomy with associated oophorectomy. It should be noted that two women with potential fertility impairment have not been included in the calculations; one who does not have or had reproductive desire and another young woman whose fertility has not been considered to be affected because she has not yet sought for pregnancy. Both women have greatly decreased AMH levels for their age, below the 5th percentile, and in case they had sought for pregnancy, they probably would have found difficult to fulfil their reproductive desire and the percentage of women with a desire for fertility affected would amount up to 22.5%.

In view of the results, we think that the group of patients who should receive iodine must be very well selected, especially in low-risk tumours, and when deciding on treatment with I^131^ it is important to inform about the possible consequences that I^131^ might take on fertility and to perform reproductive counselling. A good option for patients who would like to have offspring in the future and have not yet fulfilled their desire for reproduction could be to carry out an ovarian reserve study. Antimüllerian hormone determination in these patients is adequate to assess their follicular reserve^15,16^. In those patients with a compromised ovarian reserve, as recommended by the ASCO (American Society of Clinical Oncology), possible interventions aimed at preserving fertility, such as oocytes cryopreservation^26,27^, could be considered. Going a step further, those patients we have in follow-up after the diagnosis and treatment of a differentiated thyroid carcinoma, should be asked about their future reproductive desire, since even if they have already received the treatment with radioactive iodine, it may be convenient to advance the search for gestation or to preserve oocytes before an irreversible deterioration can occur.

## Supplementary Information

Supplementary Information.
