# Supplementary material for: Radioactive iodine and female fertility
Source: Sci Rep. 2022 Mar 8;12:3704. doi: 10.1038/s41598-022-07592-8 (PMC8904766; doi:10.1038/s41598-022-07592-8)
Supplement: Supplementary file 1 — Supplementary Information. [file 41598_2022_7592_MOESM1_ESM.docx]

Supplementary material

Table 1. Radioiodine dosage.

| **I^131^ administration age** |  |
| --- | --- |
| Age (Mean ± SD) | 30,6 ± 7,9 years |
| Range | 15,6 – 46 years |
| **I^131^ total dosage** |  |
| Dose (Mean ± SD) | 146,8 ± 96,1 mCi |
| Range | 28 – 363 mCi |
| **Dose frequency** |  |
| I^131^ < 50 mCi | 6 women (15%) |
| I^131^ 50-99 mCi | 7 women (17,5%) |
| I^131^ ≥ 100 mCi | 27 women (67,5%) |

Table 2. Frequency of menstrual irregularities distributed by radioiodine dose.

|  | < 50 mCi de I^131^ | 50-99 mCi de I^131^ | ≥ 100 mCi de I^131^ | P |
| --- | --- | --- | --- | --- |
| Menstrual alteration | 2 | 2 | 5 | 0,326 |
